# Dermoscopy of skin metastases in advanced cancer—systemic (visceral, hematologic) and cutaneous

**DOI:** 10.3389/fmed.2024.1445811

**Published:** 2024-07-30

**Authors:** Olga Simionescu, Marian Petrică, Alina Mariana Avram, Mariana Costache, Lucian G. Scurtu, Sorin Ioan Tudorache, Polixenia Georgeta Iorga, Mariana Grigore

**Affiliations:** ^1^Faculty of Medicine, “Carol Davila” University of Medicine and Pharmacy, Bucharest, Romania; ^2^Department of Dermatology I, Colentina Clinical Hospital, Bucharest, Romania; ^3^Department of Partial Differential Equations and Applications in Sciences, “Gheorghe Mihoc-Caius Iacob” Institute of Mathematical Statistics and Applied Mathematics of the Romanian Academy, Bucharest, Romania; ^4^Faculty of Mathematics and Computer Science, University of Bucharest, Bucharest, Romania; ^5^Department of Pathology, University Emergency Hospital, Bucharest, Romania; ^6^Department of Preclinical Medicine, Faculty of Medicine, “Titu Maiorescu” University, Bucharest, Romania; ^7^Department of Medical Oncology, Bucharest Emergency University Hospital, Bucharest, Romania

**Keywords:** cutaneous metastases, skin metastases, skin cancer, dermoscopy, diagnosis, metastases

## Abstract

**Introduction:**

Skin metastases arise in 10% of cancer patients, but standardized dermoscopy diagnostic criteria for skin metastases remain poor. This study's objective was to analyze the dermoscopy features of skin metastases from advanced systemic and cutaneous cancers.

**Methods:**

A retrospective study on 715 dermoscopy images of skin metastases from 33 patients with various primary cancers (breast, ovary, melanoma, non-melanoma skin cancer, and chronic leukemia) attending two academic centers between 2013 and 2023 was performed. Four independent observers blindly analyzed patterns, colors, vessels, and elementary lesions for each metastasis (30 parameters in total).

**Results:**

The structureless white pattern was the most prominent indicator of cutaneous metastasis (81.26%, *p* < 0.001). Regardless of the primary tumor, colors pink, red, white, and tan were identified. Elementary lesions were infrequent, except for melanoma metastases that displayed dots (13.23%) and globules (11.11%). Breast cancer metastases presented: blue (41.48%) and red (34.32%) colors, irregular vessels (13.58%), and a blue-naevus pattern (22.22%). Melanoma metastases displayed: a blue-naevus pattern (61.38%), a blue color (85.71%), and a structureless-blue combination pattern (79.37%). Non-melanoma skin cancer metastases were characterized by vascular (42.11%) and angioma-like (31.58%) patterns, pink (57.89%) and red (57.89%) colors, irregular (57.89%), thin hairpin (47.37%), comma (47, 37%), and thick hairpin (26, 32%) vessels and a red, white and irregular vessels combination pattern (52, 63%). A pink structureless combination pattern was frequent (61.05%) in chronic leukemia metastases. Ovarian cancer metastases displayed a white and tan structureless combination pattern (100%) and frequently had dotted vessels (42.85%).

**Conclusion:**

Papules and nodules with a white structureless pattern suggest skin metastases, regardless of the primary tumor. A blue structureless lesion is indicative of melanoma metastasis and a vascular pattern with irregular vessels indicates a non-melanoma skin cancer metastasis. Dermoscopy stands as a reliable non-invasive diagnostic method for suspected cutaneous metastases in patients with a known cancer history.

## 1 Introduction

Cutaneous metastases (CMs) are rare (1 to 10% of all cancer patients), but they always herald advanced disease and poor prognosis (1). Conflicting data exist regarding the connection between the type of cancer and skin as a target for metastatic involvement. Many studies have indicated breast cancer as the main source of CMs (2, 3). Lung, gastrointestinal, and gynecological cancers are also important sources of secondary skin involvement (4). Up to now, many reports have focused only on internal tumors. Melanoma stands, in both sexes, as one of the main cancers to metastasize in the skin (5, 6), with an occurrence rate ranging from 10% to 17% (7).

Commonly, patients already carry a cancer diagnosis and the secondary skin involvement comes in the context of disseminated disease (5). CMs can also indicate relapse after a successful remission period. Melanoma and breast cancer are well known for delayed CMs, even after many years of stable disease. In up to one-third (10%−30%) of cases, secondary skin involvement can be the presenting sign of an underlying malignant disease (8).

CMs display a polymorphous clinical presentation, making the diagnosis challenging, especially in the absence of an underlying cancer diagnosis (9). Red, pink, skin-colored, bluish, or even pigmented papules, nodules, plaques or ulcers, and solitary or multiple lesions, are all possible clinical features of secondary skin involvement (10). Dermoscopy is a useful and easy-to-use diagnostic tool in various skin pathologies, mainly in the tumoral field, but standardized criteria for the recognition of skin metastases are still lacking (11). There is limited data available regarding reliable dermoscopic patterns in CMs and most of the information is provided by single case reports and small case series, particularly for internal cancers (10, 12–14). There are slightly more reports on dermoscopy in CMs of melanoma (15–17), but clear dermoscopy criteria are not generally used.

The most common dermoscopic finding reported in CMs of visceral cancers is the vascular pattern, described as irregular and polymorphic vessels (13). Structureless areas, either pink or white were also described (13, 18, 19). In breast cancer, a pigmented pattern mimicking melanoma was also reported (13, 20). Even in the case of melanoma CMs, there are no unified dermoscopy patterns for diagnosis. Costa et al. proposed five dermoscopic patterns with good interobserver accuracy: blue naevus-like, naevus-like, angioma-like, vascular, and unspecific (17). Aviles-Izquierdo et al. found that 75% of melanoma secondary skin involvement has a monochromatic pattern, either blue, pink, or brown (15). Other studies suggested that a vascular pattern, either atypical and polymorphic vessels or angioma-like, is the most prevalent dermoscopic indicator in melanoma CMs (16, 21).

We sought to examine patterns, colors, vessels, and elementary lesions on a large number of skin metastases from patients with different types of primary cancers, both systemic (visceral and hematologic) and primary skin cancers (melanoma and non-melanoma), and establish an accurate and easy to recognize dermoscopy pattern for cutaneous metastases.

## 2 Materials and methods

We conducted a retrospective study on 715 high-quality digital dermoscopy images (Heine Delta 20+, Nikon D90, Heine SLR photo adaptor for Nikon) of 715 skin metastases collected from 33 patients with different types of advanced primary cancers between 2013–2023 in two academic centers. All patients had a documented cancer diagnosis and skin metastases were biopsied for confirmation (in cases of multiple clinically similar lesions on the same patient only one lesion was biopsied). Pathology examination and immunohistochemical tests were used for diagnosis. We included patients with different types of primary cancers: visceral cancers (breast, ovarian), skin cancers (melanoma, squamous cell carcinoma, and Merkel carcinoma), and hematologic cancers (chronic lymphocytic and myelocytic leukemia).

**Figure 1:**
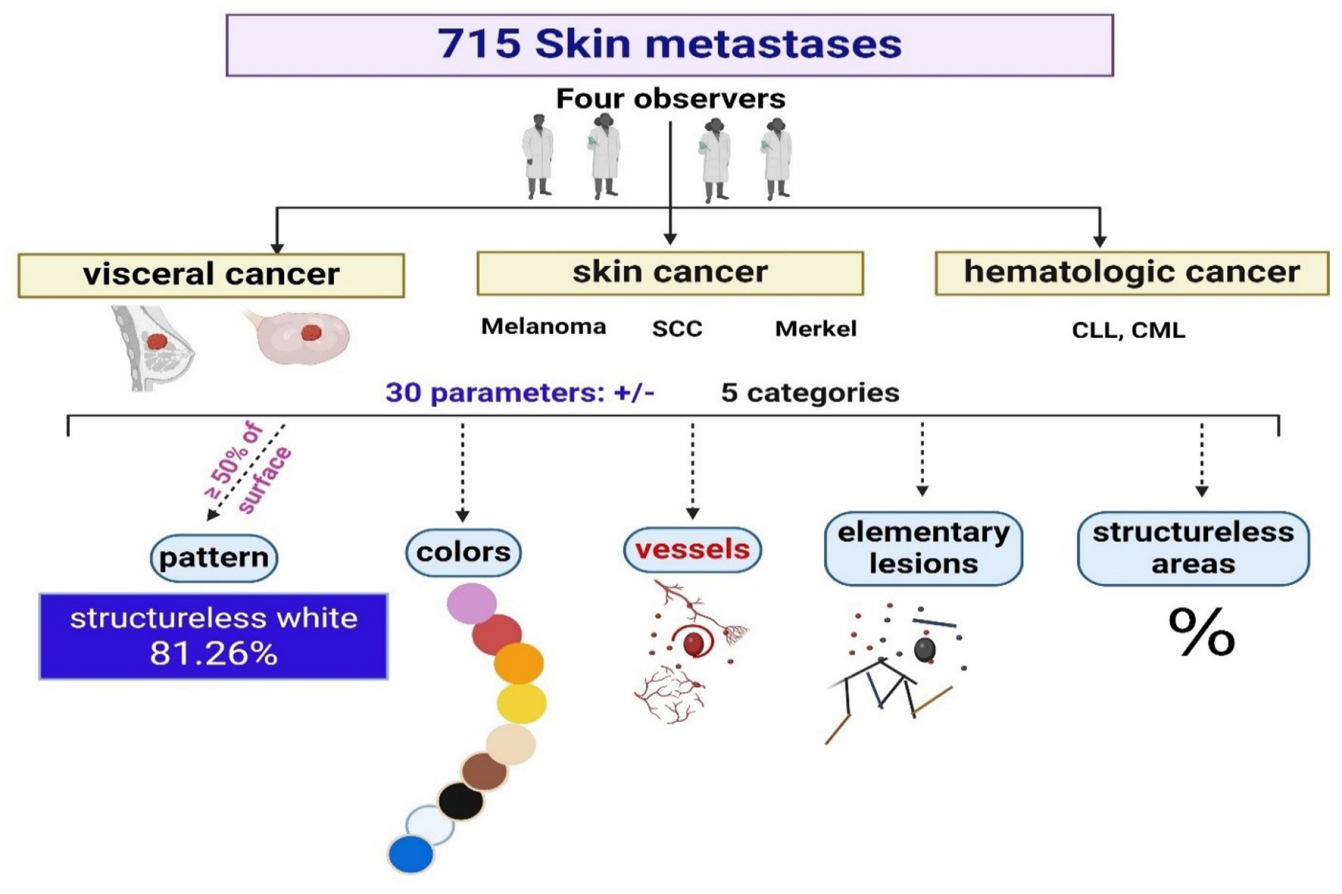
The methodology of dermoscopy image analysis of the CMs.

**Figure 2:**
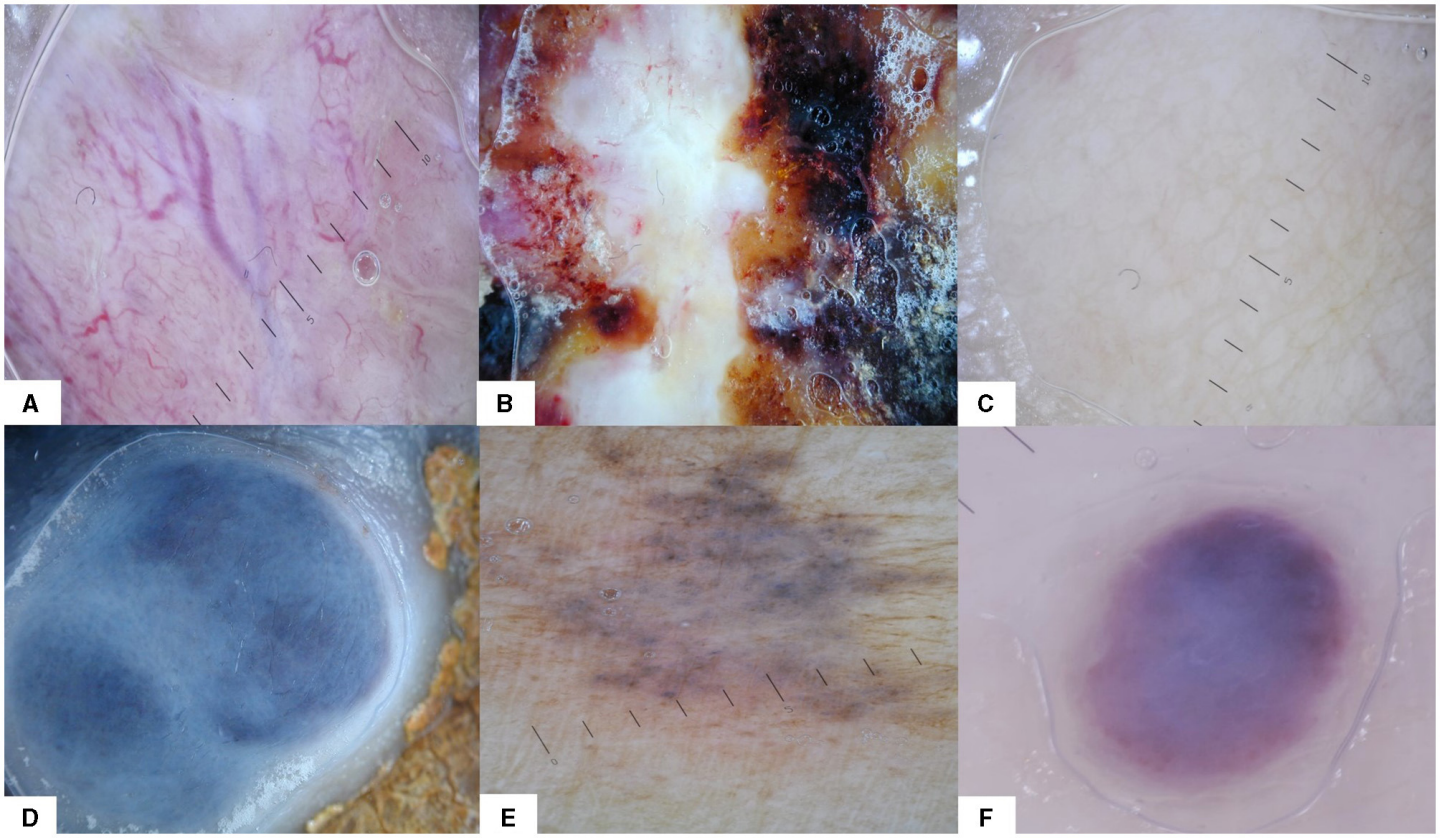
The six dermoscopy patterns that were used for analysis: **(A)** vascular, **(B)** heterogeneous, **(C)** structureless, **(D)** blue naevus-like, **(E)** naevus-like, and **(F)** angioma-like.

Four independent observers (three experts and one beginner) performed a blind image analysis on all dermoscopy images (Figure 1). None of the examiners were aware of the type of cancer it originated from. Each skin metastasis was assigned a number and was visualized and analyzed individually and on its entire surface. Each examiner performed image analysis on their device (laptop screen, tablet screen, and PC screen). Thirty parameters were included in the analysis, divided into five categories:

a. Pattern: vascular (presence of vessels, irrespective of their type), heterogenous (different colors and/or structures on the same lesion, without meeting the criteria for other patterns, and unspecific pattern), structureless (total lack of elementary lesions and vessels, irrespective of color), blue naevus-like, naevus-like (brown pigmentation, brown to black dots and globules, pigment network), angioma-like (Figure 2);

b. Colors: pink, red, orange, yellow, tan, brown, black, white, and blue;

c. Vessels: irregular, serpiginous, thin hair-pin, thick hair-pin, corkscrew, comma, dotted, and arborizing;

d. Elementary lesions- were considered the primary dermoscopic features or structures: dots, globules, lines, streaks, lacunae, and milky-white areas;

e. Percentage of structureless area.

All parameters were evaluated as present (1) or absent (0) for each metastasis, except the percentage of structureless area which was approximated with a value from 0 to 100%. If a pattern had been visible on at least 50% of the lesion surface, it would be evaluated as present. Colors, vessels, and elementary lesions were assessed as present if visible on the lesion, disregarding their surface.

All investigative data were collected into a central database (Microsoft Excel) by the study coordinator. The statistical analysis was completed using the R software. Descriptive and graphical analysis was used to check assumptions of normality and linearity for all study variables. Clinical and demographic characteristics were compared between patients with different types of cancer.

The normality of the distributions was tested rigorously by the Shapiro–Wilk Test and the symmetry of the non-normal distributions was analyzed by looking at the skewness and kurtosis indicators. Regarding the age of the patients, the *p-*value is inferior to 0.05 (*p-*value = 0.0274), therefore, the distribution of the given data is different from the normal distribution significantly (kurtosis: 1.34, skewness: −0.90). Differences between various subgroups were analyzed using the Chi-Square Test (χ2) with/without Yates' continuity correction, Fisher's Exact Test, Fisher's Exact Test with simulated *p-*value (based on 1e+08 replicates), McNemar's Test, Cochran's Q test or proportions tests where applicable. All the above tests were used with/without random method. They were also used in the subgroup analysis and for pairwise differences of the ratios, where we assessed whether there were statistical differences between metastases from different cancers with respect to different features, as described above, with/without adjustments such as scaling or randomization. Lastly, the Kruskal–Wallis Test was employed to assess the difference in the percentage of structureless areas of different types of cancer. Linear relations with a *p-*value (two-sided) ≤ 0.05 were considered significant.

## 3 Results

### 3.1 Patients

This study was conducted on 715 high-quality dermoscopy images collected from 33 patients (25 women and 8 men) with biopsy and immunohistochemical proven CMs. The mean age at the time of diagnosis was 64.78 years, with a standard deviation of 11 and a median of 68 years (range, 32–84 years). Fourteen patients were diagnosed with melanoma, 13 patients had breast cancer, 3 patients had non-melanoma skin cancers (2 squamous cell carcinoma, 1 Merkel carcinoma), 2 patients had chronic leukemia (1 lymphocytic, 1 myelocytic), and 1 patient had ovarian cancer. Most patients had multiple CMs. We analyzed 405 breast cancer CMs, 189 melanoma CMs, 19 non-melanoma skin cancer CMs, 7 ovarian cancer CMs, and 95 chronic leukemia CMs.

### 3.2 Interobserver agreement

**Figure 3:**
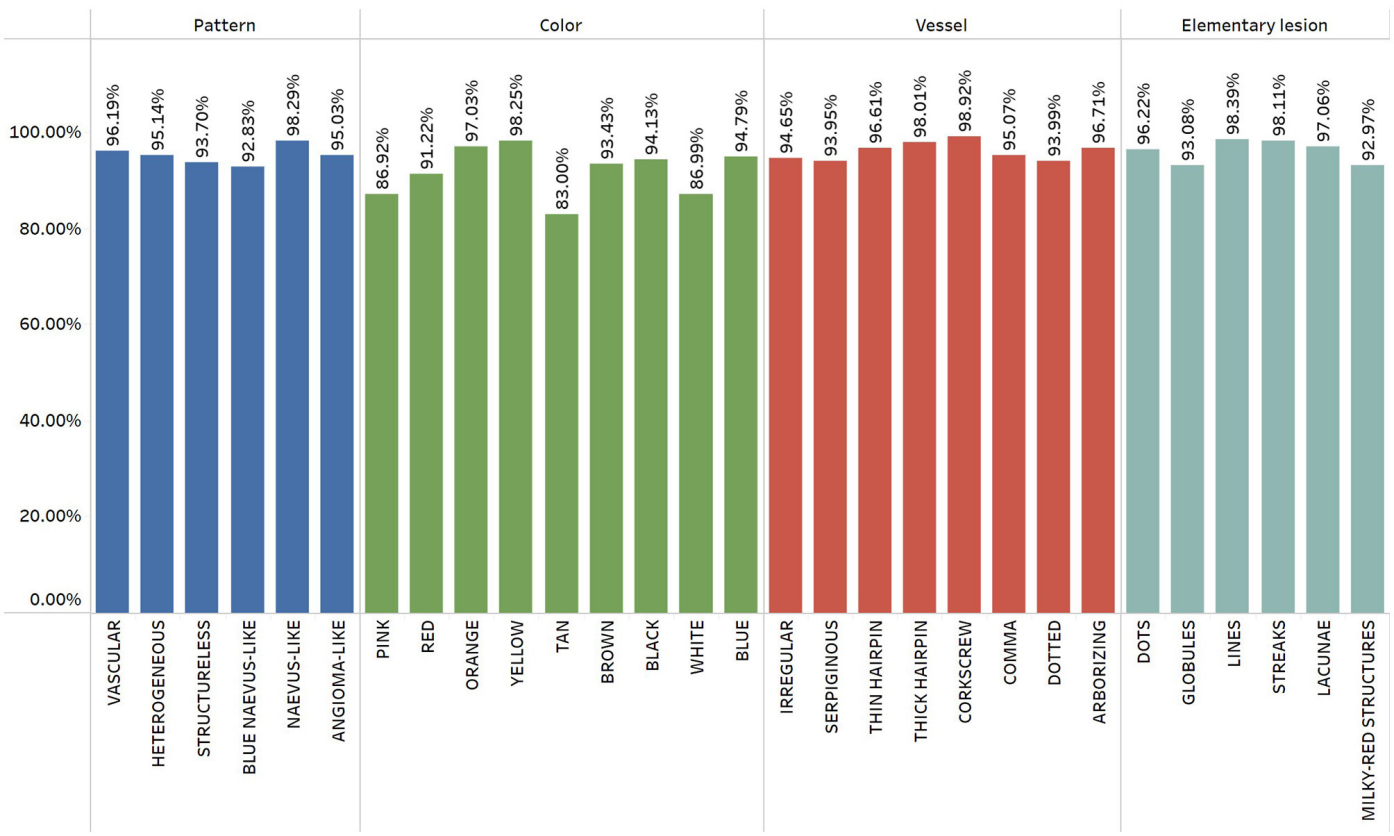
Interobserver agreement for all parameters.

The average percentage of agreement between observers for each pattern individually was >90% for all the features, except the pink color, where it was 86.29%, tan color (83.00%), and white color (86.99%) (Figure 3). The average percentage of agreement for pattern was 95.2%, while the average percentage of agreement for color was 91.75%. Furthermore, there was a high agreement regarding the presence of vessels (95.99%) and elementary lesions (95.97%).

### 3.3 Analysis results

As seen in Table 1, a structureless pattern was the dominant feature for CMs in our study (95.8%), for all types of primary cancers. Percentage of structureless areas had a mean value of 94.79 % of the surface of the lesion, for all types of metastases. Most lesions (99.57%) had only one pattern (56.22%) or two dominant patterns (43.45%).

**Table 1:** Distribution of patterns, colors, vessels, and elementary lesions in the CMs.

		**Total (*n =* 715)**	**Breast cancer (*n =* 405)**	**Melanoma (*n =* 189)**	**Non-melanoma skin cancer (*n =* 19)**	**Ovarian cancer (*n =* 7)**	**Chronic leukemia (*n =* 95)**
Pattern	Vascular	37 (5.17%)	27 (6.67%)	1 (0.53%)	8 (42.11%)	0 (0.00%)	1 (1.05%)
	Heterogeneous	50 (6.99%)	41 (10.12%)	9 (4.76%)	0 (0.00%)	0 (0.00%)	0 (0.00%)
	Structureless	685 (95.80%)	396 (97.78%)	172 (91.01%)	16 (84.21%)	7 (100.00%)	94 (98.95%)
	Blue naevus-like	206 (28.81%)	90 (22.22%)	116 (61.38%)	0 (0.00%)	0 (0.00%)	0 (0.00%)
	Naevus-like	22 (3.08%)	1 (0.25%)	21 (11.11%)	0 (0.00%)	0 (0.00%)	0 (0.00%)
	Angioma-like	31 (4.34%)	22 (5.43%)	3 (1.59%)	6 (31.58%)	0 (0.00%)	0 (0.00%)
Color	Pink	216 (30.21%)	102 (25.19%)	41 (21.69%)	11 (57.89%)	3 (42.86%)	59 (62.11%)
	Red	232 (32.45%)	139 (34.32%)	30 (15.87%)	11 (57.89%)	3 (42.86%)	49 (51.58%)
	Orange	33 (4.62%)	25 (6.17%)	4 (2.12%)	3 (15.79%)	1 (14.29%)	0 (0.00%)
	Yellow	20 (2.80%)	17 (4.20%)	2 (1.06%)	1 (5.26%)	0 (0.00%)	0 (0.00%)
	Tan	172 (24.06%)	78 (19.26%)	42 (22.22%)	3 (15.79%)	7 (100.00%)	42 (44.21%)
	Brown	59 (8.25%)	13 (3.21%)	46 (24.34%)	0 (0.00%)	0 (0.00%)	0 (0.00%)
	Black	33 (4.62%)	13 (3.21%)	20 (10.58%)	0 (0.00%)	0 (0.00%)	0 (0.00%)
	White	602 (84.20%)	395 (97.53%)	139 (73.54%)	13 (68.42%)	7 (100.00%)	48 (50.53%)
	Blue	331 (46.29%)	168 (41.48%0	162 (85.71%)	1 (5.26%)	0 (0.00%)	0 (0.00%)
Vessels	Irregular	87 (12.17%)	55 (13.58%)	18 (9.52%)	11 (57.89%)	1 (14.29%)	2 (2.11%)
	Serpiginous	23 (3.22%)	20 (4.94%)	2 (1.06%)	1 (5.26%)	0 (0.00%)	0 (0.00%)
	Thin hair-pin	31 (4.34%)	18 (4.44%)	4 (2.12%)	9 (47.37%)	0 (0.00%)	0 (0.00%)
	Thick hair-pin	23 (3.22%)	14 (3.46%)	4 (2.12%)	5 (26.32%)	0 (0.00%)	0 (0.00%)
	Corkscrew	11 (1.54%)	2 (0.49%)	5 (2.65%)	4 (21.05%)	0 (0.00%)	0 (0.00%)
	Comma	55 (7.69%)	34 (8.40%)	10 (5.29%)	9 (47.37%)	0 (0.00%)	2 (2.11%)
	Dotted	72 (10.07%)	31 (7.65%)	33 (17.46%)	4 (21.05%)	3 (42.86%)	1 (1.05%)
	Arborizing	31 (4.34%)	27 (6.67%)	2 (1.06%)	2 (10.53%)	0 (0.00%)	0 (0.00%)
Elementary lesions	Dots	36 (5.03%)	10 (2.47%)	25 (13.23%)	0 (0.00%)	0 (0.00%)	1 (1.05%)
	Globules	23 (3.22%)	2 (0.49%)	21 (11.11%)	0 (0.00%)	0 (0.00%)	0 (0.00%)
	Lines	2 (0.28%)	0 (0.00%)	2 (1.06%)	0 (0.00%)	0 (0.00%)	0 (0.00%)
	Streaks	1 (0.14%)	0 (0.00%)	1 (0.53%)	0 (0.00%)	0 (0.00%)	0 (0.00%)
	Lacunae	5 (0.70%)	3 (0.74%)	2 (1.06%)	0 (0.00%)	0 (0.00%)	0 (0.00%)
	Milky-red structures	6 (0.84%)	5 (1.23%)	1 (0.53%)	0 (0.00%)	0 (0.00%)	0 (0.00%)

A correlation between the type of primary cancer and the dermoscopy pattern of CMs was found (*P* < 0.01). Aside from the main structureless pattern, breast cancer metastases also showed a blue naevus-like pattern (22.22%), as well as a heterogeneous pattern (10.12%), while melanoma metastases showed more frequently a blue naevus-like pattern (61.38%) and a naevus-like pattern (11.11%). Non-melanoma skin cancer metastases showed a vascular pattern (42.11%) and an angioma-like pattern (31.58%). Ovarian cancer and chronic leukemia metastases were almost exclusively structureless (100% and 98.95%).

A correlation was noticed between the type of cancer and the color of metastases (*P* < 0.01). White was overall the most prevalent color (84.20%). Breast cancer metastases were characterized by blue (41.48%) and red (34.32%) colors. Melanoma metastases were strongly defined by blue color (85.71%), while non-melanoma skin cancer metastases presented pink (57.89%) and red (57.89%) colors. The ovarian CMs presented in all cases a tan color (100%) plus pink (42.86%) and red (42.86%), while chronic leukemia had a high prevalence of pink (62.11%), red (51.58%), and tan (44.21%) colors.

Furthermore, a relationship between vessels and type of cancer (*P* < 0.01) was observed. In breast cancer metastases, irregular vessels were most prevalent (13.58%), while melanoma metastases presented both dotted (17.46%) and irregular (9.52%) vessels. Non-melanoma skin cancer metastases were characterized by a large variety of vessel types: irregular (57.89%), thin hair-pin (47.37%), comma (47.37%), and thick hair-pin (26.32%). Ovarian cancer metastases presented mainly dotted vessels (42.86%), but also irregular ones (14.29%). Regardless of the primary tumor, vessels were infrequent, but all types of skin metastases showed some irregular (12.17%) and dotted vessels (10.07%).

Finally, the elementary lesions that were analyzed varied among different types of CMs. The only CMs that presented significant elementary lesions were melanoma metastases, especially dots (13.23%) and globules (11.11%).

All combinations of two and three features were also analyzed. Table 2 illustrates the top ten combinations of two features found in our study. The structureless white pattern was the most defining feature for CMs (81.26%), irrespective of the primary cancer of origin. All other combinations, of either two or three parameters, did not meet the 50% threshold in the study group. Breast and ovarian cancer metastases presented the structureless white pattern in the highest proportion (95.56% and 100% respectively), while melanoma metastases only showed this pattern in 68.78% of cases. The most prevalent pattern for melanoma metastases was a structureless blue pattern (79.37%). Chronic leukemia metastases were characterized by a structureless pink pattern (61.05%), but also a structureless tan pattern (43.16%), pink plus red (47.37%), and red plus tan (43.16%) color combinations.

**Table 2:** The top ten two-feature combinations found in this study.

**Two feature combinations**	**Total (*n =* 715)**	**Breast cancer (*n =* 405)**	**Melanoma (*n =* 189)**	**Non-melanoma skin cancer (*n =* 19)**	**Ovarian cancer (*n =* 7)**	**Chronic leukemia (*n =* 95)**
Structureless pattern + white color	581 (81.26%)	387 (95.56%)	130 (68.78%)	10 (52.63%)	7 (100.00%)	47 (49.47%)
Structureless pattern + blue color	319 (44.62%)	168 (41.48%)	150 (79.37%)	1 (5.26%)	0 (0.00%)	0 (0.00%)
White color + blue color	288 (40.28%)	168 (41.48%)	119 (62.96%)	1 (5.26%)	0 (0.00%)	0 (0.00%)
Structureless pattern + red color	216 (30.21%)	130 (32.10%)	27 (14.29%)	8 (42.11%)	3 (42.86%)	48 (50.53%)
Blue naevus-like pattern + blue color	205 (28.67%)	90 (22.22%)	115 (60.85%)	0 (0.00%)	0 (0.00%)	0 (0.00%)
Structureless pattern + blue naevus-like pattern	205 (28.67%)	90 (22.22%)	115 (60.85%)	0 (0.00%)	0 (0.00%)	0 (0.00%)
Structureless pattern + pink color	201 (28.11%)	94 (23.21%)	38 (20.11%)	8 (42.11%)	3 (42.86%)	58 (61.05%)
Blue naevus-like pattern + white color	177 (24.76%)	90 (22.22%)	87 (46.03%)	0 (0.00%)	0 (0.00%)	0 (0.00%)
Red color + white color	173 (24.20%)	132 (32.59%)	26 (13.76%)	10 (52.63%)	3 (42.86%)	2 (2.11%)
Pink color + white color	154 (21.54%)	93 (22.96%)	35 (18.52%)	7 (36.84%)	3 (42.86%)	16 (16.84%)

As shown in Table 3, not any three-feature combination was highly suggestive for all CMs, but a white and tan structureless pattern was indicative of ovarian cancer metastases (100%), while a white and blue structureless pattern (60.32%), as well as a structureless-blue naevus-like-blue pattern (60.32%), were significantly prevalent in melanoma metastases. Non-melanoma skin cancer metastases showed red and white with irregular vessel combinations in half of the metastases (52.63%).

**Table 3:** The main three feature combination patterns in CMs for different types of cancers in our study.

**Three feature combinations**	**Total (*n =* 715)**	**Breast cancer (*n =* 405)**	**Melanoma (*n =* 189)**	**Non-melanoma skin cancer (*n =* 19)**	**Ovarian cancer (*n =* 7)**	**Chronic leukemia (*n =* 95)**
Structureless pattern + white color + blue color	283 (39.58%)	168 (41.48%)	114 (60.32%)	1 (5.26%)	0 (0.00%)	0 (0.00%)
Structureless pattern + blue naevus-like pattern + blue color	204 (28.53%)	90 (22.22%)	114 (60.32%)	0 (0.00%)	0 (0.00%)	0 (0.00%)
Structureless pattern + white color + red color	159 (22.24%)	124 (36.84%)	24 (12.70%)	7 (36.84%)	3 (42.86%)	1 (1.05%)
Structureless pattern + pink color + red color	134 (18.74%)	71 (17.53%)	14 (7.41%)	2 (10.53%)	3 (42.86%)	44 (46.32%)
Structureless pattern + white color + tan color	120 (16.84%)	72 (17.78%)	23 (12.17%)	2 (12.17%)	7 (100%)	16 (16.84%)
Structureless pattern + pink color + tan color	99 (13.85%)	39 (9.63%)	16 (8.47%)	1 (5.26%)	3 (42.86%)	40 (40.11%)
Red color + white color + irregular vessels	68 (9.51%)	46 (11.36%)	9 (4.76%)	10 (52.63%)	1 (14.29%)	2 (2.11%)

## 4 Discussion

CMs represent infrequent but serious events in dermatology. They display an array of clinical features and their diagnosis can be challenging, both in solitary lesions and patients with unknown cancer history (3). Dermoscopy is an indispensable non-invasive diagnostic tool. Despite the existing clear criteria for many skin tumors, information regarding the dermoscopy features of CMs is inconsistent (22).

This paper analyzed a high number of skin metastases (715) from 33 patients with different types of advanced cancers, systemic (visceral and hematologic), and cutaneous (melanoma and non-melanoma). Thirty dermoscopy parameters (patterns, colors, vessels, and elementary lesions) were analyzed by four independent observers. Interobserver agreement was high (94.51%), although each observer analyzed the images on a different type of screen (laptop, PC, and tablet), with various display settings. The analysis parameters are acknowledged dermoscopy patterns, elementary lesions, and simple colors, widely used in routine dermoscopy. They are easy to recognize and any physician with dermoscopy training can use them in the diagnosis of CMs.

Statistical analysis was performed for all parameters independently, but also in combinations of two and three parameters. A common pattern for all skin metastases was investigated, but also the differences in dermoscopy features for CMs of different types of primary cancers.

**Figure 4:**
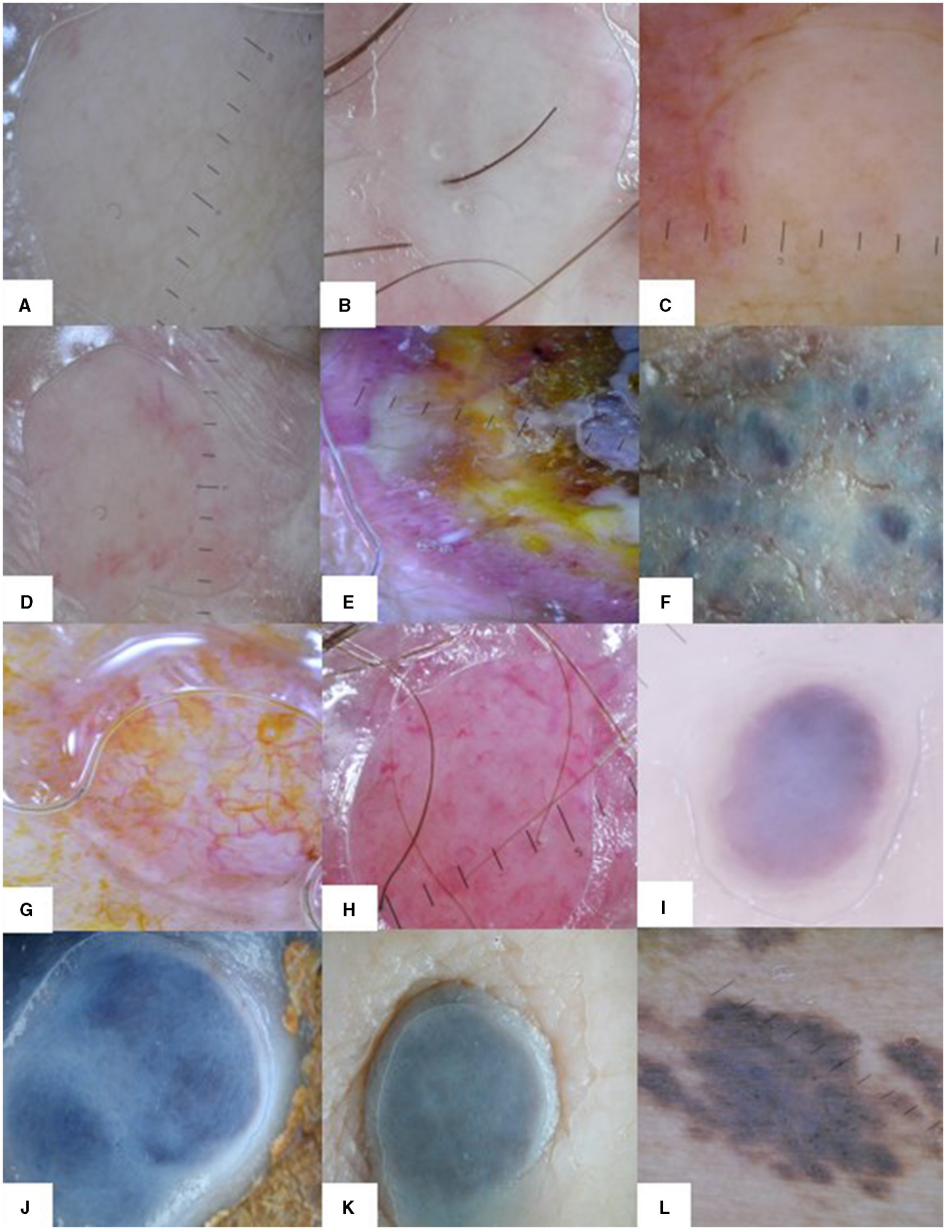
Dermoscopy patterns in CMs. The white structureless pattern in CMs of different types of primary cancers: **(A)** breast cancer, **(B)** melanoma, **(C)** chronic leukemia. Dermoscopy patterns in breast cancer CMs: **(D)** structureless white and red pattern, **(E)** heterogeneous pattern, **(F)** structureless white and blue pattern. Dermoscopy patterns in squamous cell carcinoma **(G)** and Merkel cell carcinoma **(H)** CMs: vascular pattern with different types of vessels. Dermoscopy patterns in melanoma CMs: **(I)** angioma-like pattern, **(J, K)** structureless blue and white pattern, and **(L)** naevus-like pattern with brown globules.

The main dermoscopy finding of CMs was a structureless white pattern, regardless of the primary cancer (Figures 4A–C). Most reports from the literature point out a vascular pattern as the main feature in skin metastases (11). In our analysis, a vascular pattern was described if numerous vessels were visible on at least half of the surface of the lesion, irrespective of the type of vessel. A diffuse pink color or few scattered vessels were not considered a vascular pattern and the presence of vascular lacunae was considered an angioma-like pattern.

In a case series of breast cancer patients with CMs, Chernoff et al. found 17 out of 20 lesions showed a vascular pattern, but the vascular pattern was described as either “discrete vessels (15 out of 17 patients)” or “pink homogenous structureless areas (2 out of 17 patients)” (13). The remaining three cases had various melanocytic patterns. In our study breast cancer CMs presented a predominantly structureless white pattern (95.56%), but blue (41.48%) and red (34.32%) colors were also present (Figures 4A, D–F). Irregular vessels were the most important vascular feature for breast cancer CMs in this study but were found in just over 13% of cases. A blue naevus-like pattern was evident in 22.22% of cases. Structureless white and blue (41.48%) and structureless white and red (30.63%) patterns were the most frequent combination patterns in breast cancer CMs. A few case reports on darker skin types (Moroccan, Indian) with breast cancer CMs showed a polymorphous dermoscopic presentation with multiple colors, yellow areas, polymorphic vessels, but also white and red (erythematous) structureless areas (23, 24).

A predominant vascular pattern was found only in non-melanoma skin cancer metastases (42.11%), which also showed a higher angioma-like pattern (31.58%) compared to the other skin metastases (Figures 4G–I). Non-melanoma skin cancer metastases were the most vascularized CMs in our study and presented the most varied types of vessels. Irregular vessels were the most common (57.89%), but thin hair-pin (47.37%), comma (47.37%), and thick hair-pin (26.32%) vessels were also found. Subsequently, pink (57.89%) and red (57.89%) colors were most frequent in this type of CMs, as were structureless white (52.63%), structureless pink (42.11%), and structureless red (42.11%) patterns. Half of non-melanoma skin cancer metastases presented red and white (52.63%) or red, white, and irregular vessels (52.63%) combination pattern. Until present, there are no large case series studies focused specifically on dermoscopy features of cutaneous metastases of skin carcinomas, but many single case reports of skin metastases in different internal solid organ carcinomas [colon (10), gastrointestinal (12), renal (14), nasopharyngeal (18), hepatocarcinoma (19), and prostate (25)] showed a vascular pattern, specifically with irregular vessels, but also a variety of other different types of vessels, similar to our results.

The prevalence of vessels and vascular patterns in skin metastases may be linked to angiogenesis, a key process for cancer progression and distant metastasis (25). Tumor-associated capillaries are known to be abnormal, usually tortuous, malformed, and hyperplastic (26). Also, carcinomas are known to have the second highest microvessel density within tumors (27), which could explain the predominance of vascular patterns and the variety of vessels seen in non-melanoma skin cancer metastases in our study. However, the main pattern found in our study was a structureless white pattern. Morphologically, cutaneous metastases usually spare the epidermis and are mainly dermal “bottom-heavy” infiltrates (28). Sparing of the epidermis may lead to the lack of specific focal structures and vessels on dermoscopy examination, especially in younger lesions. In this study, focal structures (dots, globules, lines, streaks, lacunae, and milky-red structures) were scarce and did not show significant differences between different types of skin metastases.

Melanoma is one of the main cancers to metastasize into the skin (6). Several classifications for dermoscopy patterns in melanoma CMs have been proposed, but there are still no generally accepted diagnostic criteria. Aviles-Izquierdo et al. described four color-based patterns: blue, pink, brown, and mixed, and reported that 75% of the 150 melanoma CMs analyzed showed a monochromatic pattern, while light brown peripheric halo, peripheral gray spots, and atypical polymorphic vessels were the most significant focal dermoscopy structures (15). Bono et al. evaluated nine dermoscopy features (homogenous, saccular, amelanotic, polymorphic and vascular patterns, color, perilesional erythema, pigmentary halo, and peripheral gray spots) on 130 melanoma CMs and found that the saccular and vascular patterns, with polymorphic atypical vessels and winding vessels, as well as pigmentary halo and peripheral gray spots, were the most significant findings (16). Costa et al. proposed a classification with a good interobserver agreement with five dermoscopy patterns: blue naevus-like, naevus-like, angioma-like, vascular, and unspecific (17). A case report of three patients suggested peripheral stellate telangiectasia as a dermoscopic clue for melanoma CMs, while other case reports indicated an angioma-like pattern (21, 29).

Our study showed most frequently a structureless blue and white pattern (60.32%) in melanoma CMs (Figures 4B, J, K). A naevus-like pattern was also present in 11.11% of cases (Figure 4L). The naevus-like skin metastases display dermal metastatic cells, forming nests, and scarcely resemble the dermo-epidermal distribution of the nevi, as metastatic cells undergo lymphatic and hematologic dissemination into the soft tissue (1, 17). Blue (85.71%) and white (73.54%) were the predominant colors and blue color was significantly more associated with melanoma CMs than other skin metastases. We did not find vascular and angioma-like patterns in melanoma CMs, but dotted (17.46%) and irregular (9.52%) vessels were noted. Melanoma CMs were the only skin metastases in our study with significant focal structures, especially dots (13.23%) and globules (11.11%). Dots correspond to melanin deposited in different layers of the skin, while globules are the result of melanocytic nests in the lower epidermis, dermal-epidermal junction, or papillary dermis (30). We attribute the scarcity of elementary lesions in melanoma CMs possibly to the dermal architecture of skin metastases with the usual sparing of the epidermis (28). Additionally, the blue color in dermoscopy suggests melanin localized in the dermis (31) and it explains the predominance of blue color in melanoma CMs.

No pattern is a certain indicator of melanoma CMs, but rather different dermoscopy patterns can raise suspicion of diagnosis (22). The polymorphism of the available data could be due to many factors, such as differences in methodology and the way a pattern is defined, but also in the age of the lesions, the type and stage of the primary melanoma, and even the skin type of the patients (32–34). Using the Bono classification, Kostaki et al. showed a correlation between dermoscopy patterns of melanoma CMs and the Breslow index of the primary melanoma (34). Homogenous and saccular patterns were the most common patterns in their study overall, but homogenous pattern was more frequent among superficial spreading melanomas, saccular pattern was prevalent among thin (>1 mm) and medium depth (1–2 mm) melanomas, while vascular pattern was observed only in skin metastases from melanomas with Breslow index of 2–4 mm. In amelanotic melanoma CMs, the most reported features are vascular structures, especially serpentine, glomerular, irregular hairpin, and corkscrew vessels (35).

Two patients with chronic leukemia (one lymphocytic, one myelocytic) with 95 metastatic skin lesions were included in our study. There is only one report in the literature on dermoscopy features in leukemia cutis (36). Sławińska et al. found a polymorphic vascular pattern with dotted vessels, linear curved vessels, and linear vessels with branches in 4 out of the 5 patients studied. In the remaining case, dermoscopy showed a diffuse pink–brownish structureless area. Our results showed a structureless pattern (98.95%) in the majority of leukemia cutis lesions with different colors associated: pink (62.11%), red (51.58%), white (50.53%), and tan (44.21%). We did not find elementary lesions in leukemia cutis lesions, nor a vascular pattern. Comma and dotted vessels were rarely present. The most suggestive combination patterns were structureless red and pink (46.32%) and structureless pink and tan (42.11%) patterns.

**Figure 5:**
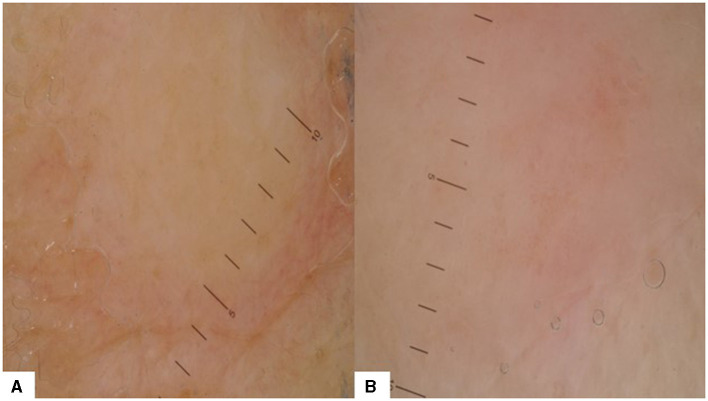
Dermoscopy patterns in CMs of ovarian cancer: **(A)** structureless white and tan pattern without vessels or elementary lesions; **(B)** structureless white, pink, and tan pattern with dotted vessels.

CMs in ovarian cancer are very rare, but they are more frequently the first sign of the neoplastic disease compared to other cancers and early diagnosis is essential (37). The only dermoscopy pattern reported for ovarian cancer CMs comes from a case report of a subungual metastasis that initially showed discrete pink and red colors, before transforming into a hemorrhagic pattern (38). One patient with ovarian cancer with seven skin metastases (non-Sister Joseph nodules) was included in our study (Figure 5). Dermoscopy showed a structureless white and tan pattern (100%) in all lesions. Red (42.86%) and pink (42.86%) colors were also present. Dotted vessels (42.86%) were predominant, but irregular vessels (14.29%) were also noted.

One case of neuroendocrine malignancy (a Merkel cell carcinoma) with one skin metastasis was also included in our study. It showed a vascular pattern, with white, red, and pink colors and a variety of vessels: irregular, thin, and thick hairpin, comma, arborizing, and dotted (Figure 4H). Skin is not a frequent metastatic site for Merkel cell carcinoma and dermoscopy patterns for skin metastases are uncommon (39). Navarrete-Dechent et al. described six skin metastases with irregular linear and arborizing vessels on a structureless red and violet background with white areas (40).

In this study, we analyzed 715 metastases from 33 patients with various advanced cancers (visceral, hematologic, and cutaneous). Most patients had multiple skin metastases, thus creating two limitations in our research. First, we only had two chronic leukemia patients, but these patients presented 95 skin metastases, combined. We also had only one patient with ovarian cancer, with seven skin metastases. As this creates an imbalance in case distribution, we performed a second statistical analysis excluding these three patients to see the impact on our results. The main results did not change significantly.

A second limitation may result from the presence of multiple metastases in the same patient. We considered each papular or nodular skin metastasis visible on a patient's skin as an independent lesion and included it in our study. Each metastasis originates from a cancerous cell that migrates from the primary tumor, invades the dermis, and results in a secondary proliferation (41). Although diffuse infiltration of metastatic cancer cells in the dermis is possible, most secondary cutaneous lesions are nodular and papular (42, 43). There are several reports that different dermoscopy patterns may arise in the same patient with multiple skin metastases, hence individual examination is warranted (23, 44). Each skin metastasis is an individual dermal proliferation, with the usual sparing of the epidermis, although lesions may appear in crops forming a plaque (28). Epidermotropic metastases can arise, especially in melanoma, but we did not encounter such cases in our research (45). As a result of the epidermal sparing on the pathology examination, the relevant immunohistochemical tests, and the documented oncological history of all patients, only one biopsy was performed for multiple clinically similar metastases on the same patient. Furthermore, most dermoscopy studies of CMs include patients with multiple lesions (15, 16). Although it is sometimes difficult to make a clinical distinction between skin metastases and other skin tumors, including benign tumors, the distribution of multiple lesions in plaques is clinically relevant for metastatic tumors.

## 5 Conclusion

This is the first extensive report of key dermoscopy features in skin metastases from different types of primary advanced cancers, systemic (visceral, hematologic), and cutaneous (melanoma and non-melanoma skin cancer).

The structureless white pattern should raise suspicion of skin metastases and prompt further investigations, mainly in patients with known oncological history. Significant differences exist between breast cancer, melanoma, and non-melanoma skin cancer metastases. The color blue on a structureless lesion should raise suspicion for melanoma skin metastasis. The vascular pattern and irregular vessels were most indicative of skin carcinoma metastases in our study. A first-glance approach to structure and color patterns can be an effective tool for non-invasive diagnosis, easy to perform, in any dermatological practice.

## Data availability statement

The raw data supporting the conclusions of this article will be made available by the authors, without undue reservation.

## Ethics statement

The studies involving humans were approved by Ethics Committee of the University of Medicine and Pharmacy “Carol Davila” Bucharest. The studies were conducted in accordance with the local legislation and institutional requirements. The participants provided their written informed consent to participate in this study.

## Author contributions

OS: Writing – review & editing, Supervision, Resources, Methodology, Investigation, Formal analysis, Data curation, Conceptualization. MP: Writing – original draft, Methodology, Formal analysis, Data curation. AA: Writing – review & editing, Formal analysis. MC: Writing – review & editing, Data curation. LS: Writing – review & editing, Formal analysis, Data curation. ST: Writing – review & editing, Supervision, Methodology. PI: Writing – review & editing, Methodology, Data curation. MG: Writing – original draft, Supervision, Formal analysis, Data curation.
